# Bortezomib-Based Regimens for Newly Diagnosed Multiple Myeloma in China: A Report of 12-Year Real-World Data

**DOI:** 10.3389/fphar.2020.561601

**Published:** 2020-12-11

**Authors:** Jingsong He, Donghua He, Xiaoyan Han, Gaofeng Zheng, Guoqing Wei, Yi Zhao, Yang Yang, Wenjun Wu, Jiaping Fu, Lihong Shou, Hongwei Kong, He Huang, Zhen Cai

**Affiliations:** ^1^ Bone Marrow Transplantation Center, The First Affiliated Hospital, School of Medicine, Zhejiang University, Hangzhou, China; ^2^ Department of Hematology, Shaoxing People’s Hospital, Shaoxing, China; ^3^ Department of Hematology, Huzhou Central Hospital, Huzhou, China; ^4^ Department of Hematology, People’s Hospital of Quzhou City, Quzhou, China

**Keywords:** bortezomib-based regimen, new diagnosed, multiple myeloma, efficacy and survival, real-world data

## Abstract

**Background:** Improve the treatment quality might affect patients’ efficacy and survival.

**Methods:** Five hundred thirty multiple myeloma patients treated in four hematological centers in China from February 2006 to August 2018 were enrolled. General characteristics, treatment regimens and cycles, efficacy, survival and adverse events of the patients treated before and after August 2013 (later refer to as the before-2013 and after-2013 group) were analyzed and compared.

**Results:** The results suggested that patients who received optimized treatment regimen and route of administration completed more cycles of treatment in the after-2013 group. Although the overall response rate was similar between the two groups (88.6 vs. 90.5%), patients in the after-2013 group had higher complete remission rate (39.1 vs. 28.6%) and better progression-free survival. Subgroup analysis suggested that patients aged 65 years and older, with non-high-risk D-S, ISS, and R-ISS stages, had a significant benefit in progression-free survival.

**Conclusion:** Therefore, in clinical practice in China, by reducing the economic burden brought by the treatment on patients and optimizing the treatment regimen, more patients can be treated with better regimens in a prolonged duration to achieve better efficacy and survival, especially in elderly and non-high-risk patients.

## Background

Multiple myeloma (MM) is a type of hematological tumor with malignant proliferation of plasma cells, and tumor cells could produce large number of monoclonal immunoglobulins and light chain proteins. MM cells and the secreted M proteins can cause a series of clinical symptoms, such as bone destruction, hypercalcemia, anemia and renal insufficiency. Chemotherapy has been the only available treatment for the disease for many years, with an overall response rate [ORR, partial response (PR) or better efficacy] of 60–70%, a complete response (CR) rate of only about 5–8%, and median survival of about 3–5 years (Kyle et al., 2003; Palumbo and Anderson, 2011). Since 2000, with the introduction of the proteasome inhibitor bortezomib, as well as the immunomodulators thalidomide and lenalidomide, the treatment paradigm for MM has been improved. Due to the use of new drugs, the overall survival (OS) of newly diagnosed MM patients has been improved by 50% (44.8 vs. 29.9 m) (Kumar et al., 2008), among which bortezomib has become the main first-line treatment option for MM patients (Mikhael et al., 2013; Coriu et al., 2018; Mohty et al., 2018; Raab et al., 2018).

In 2006, bortezomib was approved for the treatment of MM patients in China, and has been adopted as the first-line treatment option for MM since February 2006 in our center (Zheng et al., 2009; He et al., 2014), with an ORR of up to 90%, very good PR (VGPR) or better greater than 60%. The main non-hematological adverse reactions were herpesvirus infection and peripheral neuropathy (PN), which significantly affected the clinical use of bortezomib and treatment durations (He et al., 2014; Xu et al., 2018). In addition, as bortezomib was expensive and not covered by the National Health Insurance in the early stage, most patients discontinued treatment after their disease being controlled. Since August 2013, the Chinese Cancer Foundation, with the support of Xian Janssen, has launched the VELCADE Patient Assistance Program. Patients were eligible to receive five more cycles as medication assistance after receiving four cycles of VELCADE (trade name of bortezomib), and bortezomib has been covered by the National Health Insurance since September 1, 2017.At that time, Chinese hematologists have therefore accumulated more clinical experience in the treatment of MM patients with bortezomib, and the bortezomib-based regimen has been greatly improved. For example, intravenous injection (IV) was replaced by subcutaneous injection (SC) with similar efficacy but more mild adverse reactions. For elderly patients with poor physical fitness, they are administrated once a week rather than twice a week. Moreover, in the course of treatment, patients are given full care and management, especially in the prevention of herpes virus infection and PN. In clinical practice, the improvement of treatment quality is inevitable to change the characteristics, efficacy and survival of patients who receive bortezomib-based regimen, but few clinical data could be found in the Chinese real-world. Taking the August 2013 as the cut-point, the status, efficacy, adverse reactions and survival of Chinese newly diagnosed MM patients treated with bortezomib-based regimen before and after this time point were analyzed in this study.

## Materials and Methods

### Patients

This retrospective analysis study was approved by the Ethics Committee of the First Affiliated Hospital of Zhejiang University. According to WHO or International Melanoma Working Group (IMWG) diagnostic criteria, all patients from the 4 hematological centers were newly diagnosed as symptomatic or progressive MM, with detectable M-protein in blood/urine. After being fully-informed and signing the informed consent, the patients received bortezomib-based treatment as the first line at our center, with at least one cycle of treatment completed and response assessment obtained. From Febuary 1, 2006 to July 31, 2013, 231 patients were eligible for assessment (referred to as the before-2013 group); from August 1, 2013 to July 31, 2018, 299 patients with newly diagnosed MM eligible for assessment (referred to as the after-2013 group).

All patients were assessed according to Durie-Salmon (D-S) stage and International Staging System (ISS) stage at diagnosis. Based on the preference of patients and their families, specific chromosomal abnormalities in most patients’ bone marrow plasma cells were detected by fluorescence *in situ* hybridization (FISH) with probes 17p13, 1q21, 13q14, and 14q32. For economic and technical reasons, only a small number of patients with 14q32 rearrangement were tested for specific translocations, such as t (4; 14), t (6; 14), t (11; 14) or t (14; 20). Patients were retrospectively assessed according to the revised‐ISS (R‐ISS) stage in conjunction with their ISS stage, serum lactate dehydrogenase level, and FISH results obtained.

### Treatment

All patients received bortezomib-based regimens, including doublet regimen, such as PD (it means bortezomib and dexamethasone), as well as triplet regimen based on PD, such as PCD (PD in combination with cyclophosphamide), PAD (PD in combination with doxorubicin) and PTD (PD in combination with thalidomide). Bortezomib (1.3 mg/m^2^) was administrated by IV (from February 2006 to November 2012), and by SC (after November 2012) on d1, 4, 8, and 11, with a course of treatment lasting 4 weeks. In the after-2013 group, some elderly patients aged 65 years and older were administrated with bortezomib once a week for 1 course of treatment set to 5 weeks. Dexamethasone (20 mg/day) was given following bortezomib by IV on d1–2, 4–5, 8–9 and 11–12. Doxorubicin and cyclophosphamide were administrated by IV (10 mg/m^2^ and 200 mg/m^2^, respectively) on d1–4 before August 2013, after, those were given following bortezomib on d1, 4, 8 and 11. Thalidomide was given orally before bedtime at 100 mg daily during the entire treatment cycle.

**TABLE 1:** Clinical Characteristics before primary treatment of multiple myeloma patients.

	Before 2013 N = 231	After 2013 N = 299	*p*
Age, n (%)			
Median (range)	60 (31∼83)	63 (31∼89)	0.017
<65 years	166 (71.8)	173 (57.9)	0.001
≥65 years	65 (28.1)	126 (42.1)	
Gender, n (%)			0.284
Male	142 (63.9)	170 (56.9)	
Female	89 (36.1)	129 (43.1)	
Type of myeloma, n (%)			0.023
Heavy chain			
IgA	62 (26.8)	75 (25.2)	
IgG	108 (46.8)	139 (46.5)	
IgD	4 (1.7)	23 (7.7)	
Light chain	56 (24.2)	61 (20.4)	
Biphenotypic	1 (0.4)	1 (0.3)	
LDH			0.305
Normal	190 (84.1)	241 (80.6)	
Elevated	36 (15.9)	58 (19.4)	
Unknow	5	0	
Durie-Salmon Staging, n (%)			0.084
1A + B	11 (4.8)	23 (7.7)	
2A + B	36 (15.6)	35 (11.7)	
3A	129 (55.8)	188 (62.9)	
3B	55 (23.8)	53 (17.8)	
ISS, n (%)			0.045
1	57 (24.7)	101 (33.8)	
2	82 (35.5)	83 (27.7)	
3	92 (39.8)	115 (38.5)	
R-ISS^a^			0.020
1	13 (9.0)	29 (11.1)	
2	107 (73.8)	157 (60.4)	
3	25 (17.2)	74 (28.5)	
Unknow	86	39	

a Part of patients’ condition cannot be judged because of the lack of FISH result.

According to patient’s age, performance status, and preference of the patients and their families, patients underwent autologous hematopoietic stem cell transplantation (ASCT) after achieving at least PR response with 3 and 4 cycles of induction therapy. Cyclophosphamide was administrated 1.5 g/m^2^ for 2 days in combination with G-CSF (7.5 µg/kg) to mobilize peripheral blood stem cells, with the number of stem cells obtained required to be at least 2 × 10^6^/kg. The ASCT conditioning regimen was melphalan 200 mg/m^2^, combined with 4 doses of 1 mg/m^2^ bortezomib in some patients.

After 3 and 4 cycles of induction therapy with or without ASCT, some patients received maintenance therapy. The patients in the before-2013 group mainly received thalidomide (100 mg/day) as maintenance, whereas those in the after-2013 group received bortezomib-based regimen as maintenance, with 3-month a cycle. The remaining patients received thalidomide or lenalidomide as maintenance, and a small number of patients did not receive maintenance therapy.

Some of the patients in the before-2013 group and all patients in the after-2013 group routinely received prophylactic antiviral treatment with penciclovir. Specifically, patients took famciclovir tablets (250 mg) orally twice a day for 2 weeks after chemotherapy, with applicable dose adjustment according to creatinine clearance in patients with renal insufficiency. None of the patients were routinely given anticoagulation or antiplatelet therapy.

### Response and Adverse Events Assessment

Patients’ response was evaluated based on the IMWG response assessment criteria, including complete response (CR), very good partial response (VGPR), partial response (PR), stable disease (SD), and progressive disease (PD) (Durie et al., 2006). progression-free survival (PFS) was assessed from the time of patients receiving the first cycle of treatment to disease progression, or death occurred or to the end of subsequent follow-up (censored data). OS was measured from the time of patients receiving the first cycle of treatment to death occurred or the end of subsequent follow-up. Adverse reactions were assessed at each hospital visit and treatment according to the National Cancer Institute’s Common Terminology Criteria (version 3.0; NCI-CTC 3.0).

### Statistical Analysis

Patients enrolled before 2013 were followed up until October 30, 2013, whereas those enrolled after 2013 were followed up until October 30, 2018. Patients were assessed for treatment efficacy and adverse events immediately after their completion of a cycle of treatment. Patients’ clinical characteristics, treatment regimens, and efficacy were compared. T-test was used for continuous data and chi-square test for categorical data. Kaplan-Meier was used to analyze survival curve, and log-rank test was used for survival (PFS and OS) difference analysis. All data tests were two-sided, and *p* < 0.05 were considered to be statistically significant.

## Results

### Patients Characteristics

A total of 530 patients were enrolled, including 231 before 2013 and 299 after 2013. Patients’ general characteristics are shown in Table 1. Patients treated with bortezomib-based regimen in the after-2013 group were older, with a median age of 63 years old in the after-2013 group and 60 years old in the before-2013 group. The proportion of patients over 65 years old was increased from 28.1% in the before-2013 group to 42.1% in the after-2013 group. In addition, the proportion of patients with D-S stage 3B was decreased in the after-2013 group (23.8 vs. 17.8%), while that with ISS stage I was increased in the after-2013 group (33.8 vs. 24.7%).

**TABLE 2:** Result of fluorescence *in situ* hybridization test.

n (%)	Before 2013 N = 231	After 2013 N = 299	*p*
Not available	147	61	
Available	84	238	
1q21 Amplification	36 (42.9)	141 (59.2)	0.001
17p Deletion	23 (27.4)	23 (9.7)	
13q Deletion	39 (46.4)	96 (40.3)	
IgH Rearrangement	29 (34.5)	85 (35.7)	
Non-high-risk Factors^a^	38 (45.2)	90 (37.8)	0.232
High Risk Abnormity	46 (54.8)	148 (62.2)	
One	33 (39.3)	132 (55.5)	
Double	13 (15.5)	16 (6.7)	

a High risk abnormity refers to the amplification of 1q21 or 17p deletion.

Based on patients’ pre-treatment serum lactate dehydrogenase levels, FISH test results and ISS staging, we retrospectively analyzed R-ISS staging (staging could not be achieved in 86 and 39 patients respectively in the two groups, due to lack of iFISH data). Among the 405 patients who were able to be staged, the proportion of patients in stage 3 (high-risk) was increased significantly in the after-2013 group (17.2 vs. 28.5%) (Table 2).

### Treatment Duration and Efficacy

All patients received bortezomib-based regimens, including PAD, PCD, PTD, and PD regimens, as previously described. In the before-2013 group, median completed cycles were three (1–9), with an average of 3.1. 96 (41.6%) patients completed four or more cycles, whereas only 17 (7.4%) patients completed five or more cycles of treatment. In the after-2013 group, median cycles were five (1–12), with an average of 5.4. 220 (73.6%) patients completed 4 or more cycles of treatment, and more than 50% (169) patients completed 5 or more cycles. There was a significant difference between the two groups (*p* < 0.001). Treatment regimens, cycles of treatment and efficacy data are shown in Table 3.

**TABLE 3:** Therapy and efficacy of multiple myeloma patients.

	Before 2013 N = 231	After 2013 N = 299	*p* ＜0.001
Courses			
Median (range)	4 (1∼9)	5 (1∼12)	
＜4	135 (58.4)	79 (26.4)	
≥4	96 (41.6)	220 (73.6)	
≥5	17 (7.4)	169 (56.5)	
Regimens, n (%)			＜0.001
PD	48 (20.8)	52 (17.4)	
PAD	62 (26.8)	36 (12.1)	
PCD	86 (37.2)	193 (64.5)	
PTD	35 (15.2)	18 (6.0)	
ASCT, n (%)	28 (12.1)	34 (11.4)	0.790
Response			0.013
＜PR	22 (9.5)	34 (11.4)	
PR	81 (35.1)	97 (32.4)	
VGPR	62 (26.8)	51 (17.1)	
CR	66 (28.6)	117 (39.1)	

In the after-2013 group, more patients were treated with PCD regimen (64.5 vs. 37.2%) as it has good tolerability, better efficacy and lower cost and it’s easy to use. Although more patients were over 65 years of age, the proportion of patients on the two-drug combination PD regimen decreased due to better treatment tolerability with subcutaneous bortezomib and weekly treatment schedule. Overall, 62 patients received ASCT, and all of them were under 65 years of age and had at least PR response to the previous treatment. There were 28 cases in the before-2013 group, accounting for 16.9%, and 34 (19.7%) cases in the after-2013 group. The overall efficacy analysis suggested that patients may have similar ORR (90.5 vs. 88.6%), but patients in the after-2013 group had higher CR rates (39.1 vs. 28.6%) (Table 3).

### Survival Analysis

#### Progression-Free Survival

**FIGURE 1:**
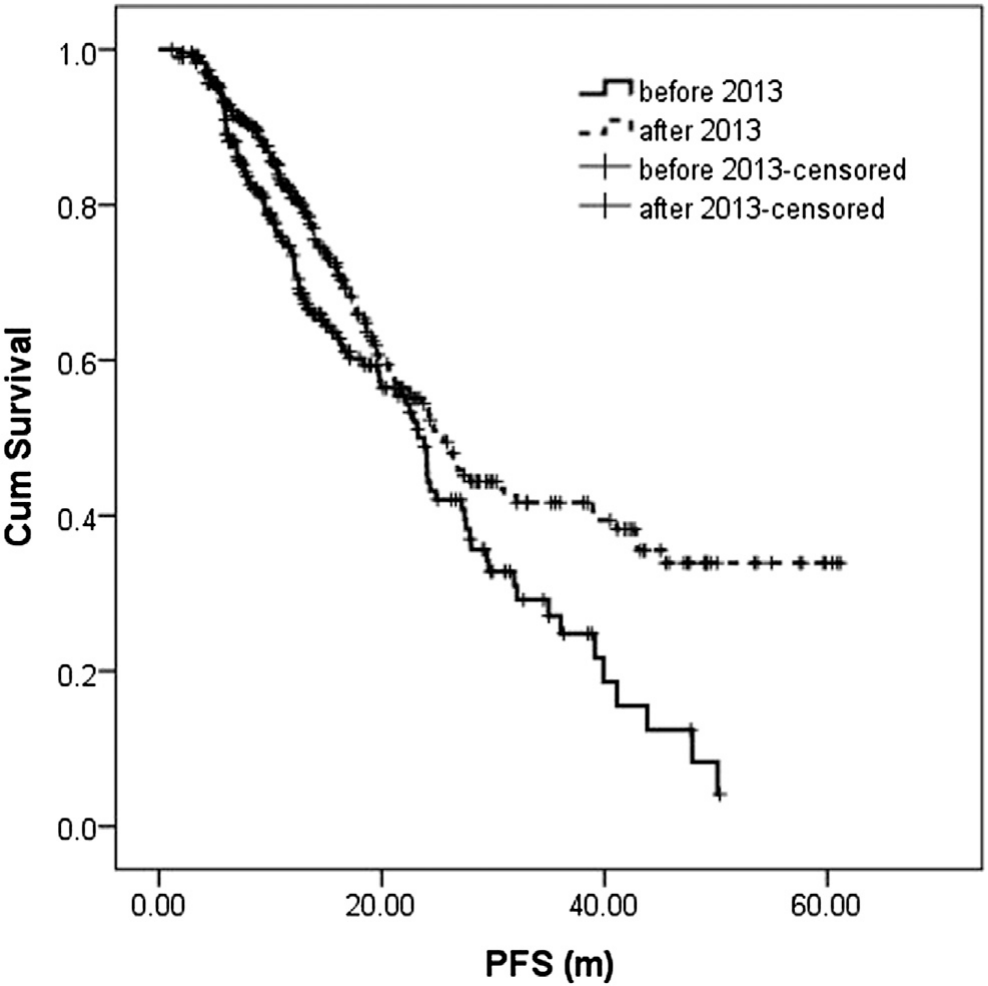
Progression-free survival (PFS) analysis and comparison between the two groups.

The median follow-up in the before-and after-2013 groups were 18.0 (1.1–68.0 m) and 20.4 m (0.9–61.5 m), respectively. During the follow-up, 106 patients (45.9%) and 122 patients (40.8%) had disease recurrence or progression in the before-and after-2013 groups with a median PFS of 23.3 m (95% CI: 21.1–25.5) and 25.5 m (95% CI: 22.0–29.1), respectively, showing statistically significant difference between two groups (*p* = 0.004). 3-year PFS was 27.1 and 41.7%, respectively (Figure 1).

#### Overall Survival

**FIGURE 2:**
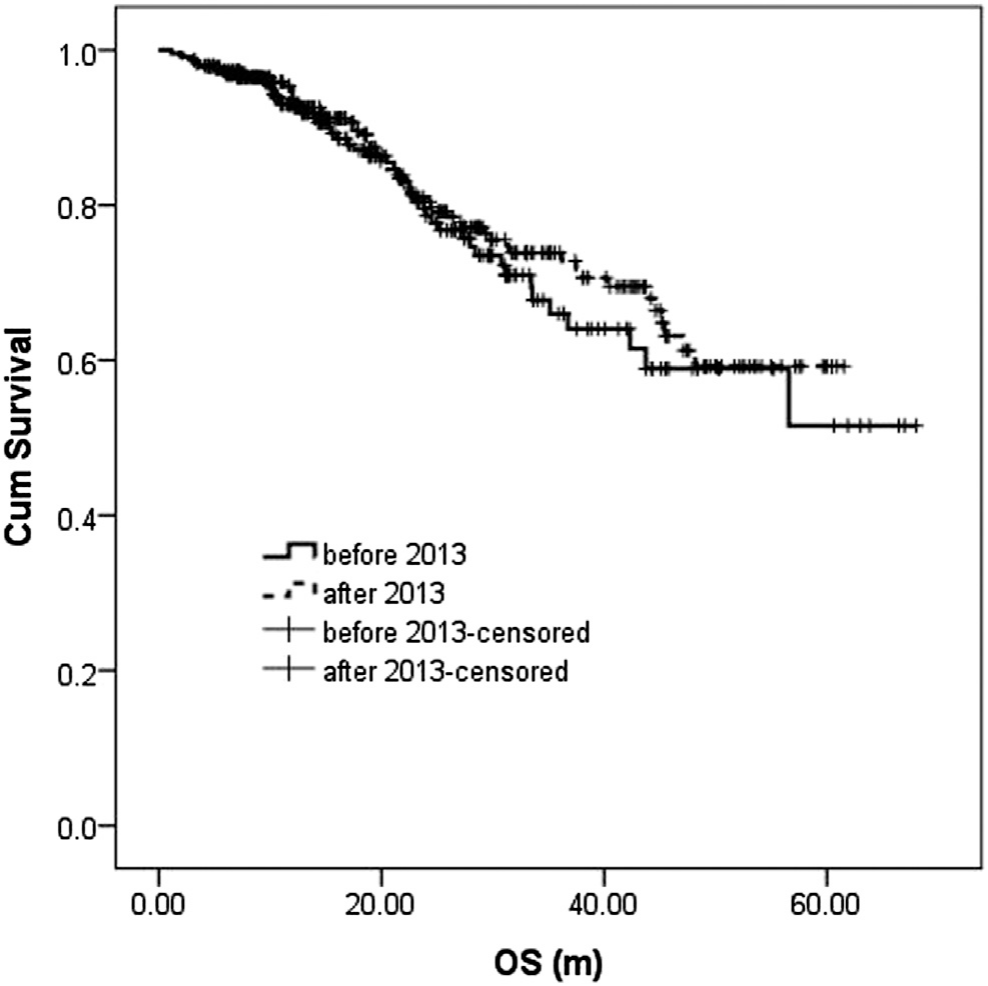
Overall survival (OS) analysis and comparison between the two groups.

During the follow-up, 47 patients (20.3%) died in the before-2013 group, including 43 died of disease progression and 4 of treatment-related severe infection. In the after-2013 group, 61 patients (20.4%) died, including 52 died of disease progression, three of severe infection, two of cerebrovascular accident, three of sudden cardiac death (possible cardiac amyloidosis indicated by cardiac ultrasound), and one of other causes. The median OS was not reached in either two groups, with no statistically significant difference (*p* = 0.489). The 5-year OS was 51.6 and 59.2%, respectively (Figure 2).

### Subgroup Analysis

#### Treatment Response

Patients who did not achieve PR response after 3–4 cycles of treatment were treated with other regimens, including thalidomide, lenalidomide, or conventional chemotherapy. Among the 474 patients who achieved PR response or better, 91/209 (43.5%) experienced disease progression in the before-2013 group and 101/265 (38.1%) in the after-2013 group. The 3-year PFS was 29.3 and 45.1%, respectively, showing statistically significant difference between the two groups (*p* = 0.003). Among 296 patients with VGPR response or better, 57/128 (44.5%) had disease progression in the before-2013 group and 56/168 (33.3%) in the after-2013 group. The 3-year PFS was 30.8 and 55.1%, respectively, showing statistically significant difference between the two groups (*p* = 0.001) (Table 4).

**TABLE 4:** Survival condition of multiple myeloma patients.

Median Survival m (95% CI)	Progression-free survival		*P*	Overall survival	
	Before 2013	After 2013		Before 2013	After 2013	*P*
Response ≥ PR (n = 474)	24.1 (20.7∼27.4)	26.8 (16.9∼36.7)	0.003	NR	NR	0.230
≥VGPR (n = 296)	24.3 (20.6∼28.0)	NR	0.001	NR	NR	0.477
Age <65 (n = 339)	27.2 (23.5∼30.9)	27.9 (21.0∼34.8)	0.145	NR	NR	0.966
≥65 (n = 191)	12.7 (9.3∼16.1)	24.1 (18.7∼29.5)	<0.001	33.4 (28.5∼38.4)	45.4 (42.1∼48.7)	0.067
Courses ＜4 (n = 214)	19.9 (12.9∼26.9)	16.1 (12.2∼20.0)	0.454	56.6 37.5 (13.4∼61.6)	NR	0.040
≥4 (n = 316)	24.3 (19.8∼28.8)	30.4 (17.8∼43.0)	0.025	NR	NR	0.561
Non-High Risk						
DS 1∼3A (n = 422)	24.3 (20.6∼28.0)	27.2 (17.2∼37.3)	0.024	NR	NR	0.533
ISS 1∼2 (n = 323)	24.3 (17.1∼31.6)	27.2 (21.0∼33.4)	0.012	56.6 (32.5∼80.6)	NR	0.096
RISS 1∼2 (n = 431)	24.0 (19.6∼26.7)	27.9 (17.4∼38.4)	0.001	NR	NR	0.086
High Risk						
DS 3B (n = 108)	12.1 (3.2∼21.0)	18.6 (14.3∼22.9)	0.119	36.7 (5.5∼58.0)	37.7 (26.5∼48.9)	0.682
ISS 3 (n = 207)	23.1 (19.6∼26.7)	20.7 (14.1∼27.2)	0.177	NR	44.0 (33.5∼54.4)	0.441
RISS 3 (n = 99)	16.3 (7.0∼25.7)	18.6 (13.8∼23.5)	0.468	23.2 (14.3∼32.2)	23.4 (15.7∼31.1)	0.717

Among 474 patients who achieved PR response or better, 37/209 (17.7%) died in the before-2013 group and 44/265 (16.6%) in the after-2013 group. Median OS was not reached (NR) for either group. 5-year OS was 53.1 and 65.8%, respectively. There was no statistical difference between the two groups (*p* = 0.230). Among 296 patients who achieved VGPR response or better, 18/128 (14.1%) patients died in the before-2013 group and 24/168 (14.3%) in the after-2013 group. The 5-year OS was 68.0 and 71.5%, respectively, with no statistical difference between the two groups (*p* = 0.477) (Table 4).

#### Age

For patients younger than 65 years, there was no significant difference in either PFS or OS between the two groups, with 3-year PFS of 30.5 and 44.8%, respectively, and 5-year OS of 58.4 and 70.5%, respectively. However, in patients aged 65 years and older, there was significant difference in PFS (*p* < 0.001), with median PFS of 12.7 and 24.1 m, and 3-year PFS of18.1 and 25.5%, respectively. OS was also superior in the after-2013 group than that in the before-2013 group (*p* = 0.067), with median OS of 45.4 and 33.4 m, respectively (Table 4). The reason may be the fact that two-thirds of elderly patients in the after-2013 group received four or more cycles of treatment, while only 30.8% in the before-2013 group (*p* < 0.001). Also, it might be benefited by the decrease of drug costs and 73/126 (57.9%) patients receiving bortezomib once per week in the after-2013 group.

#### Number of Treatment Courses

In patients who completed less than four cycles of treatment, there was no significant difference in PFS between the two groups (*p* = 0.454), with 3-year PFS of 25.4 and 22.1%, respectively. However, there was a significant difference in OS (*p* = 0.040), with 5-year OS of 48.4 and 37.1%, respectively, which was possibly due to the difference in the reasons for failure to receive adequate duration of treatment between the two groups. In the before-2013 group, about 75% (101/135) of patients discontinued treatment due to economic reasons; while in the after-2013 group, more than 50% (40/79) of the patients discontinued due to poor response (24 cases), intolerable adverse reactions (12 cases), or influence by early deaths (four cases). In patients who completed four and more cycles, there was a significant difference in PFS (*p* = 0.025), with 3-year PFS of 27.8 and 48.0%, respectively, but there was no significant difference in OS (*p* = 0.561), with 5-year OS of 62.2 and 66.4%, respectively.

#### Clinical Stages

According to D-S, ISS and R-ISS stages, a significant difference was found in the PFS for non-high-risk patients (including patients in D-S stage 1-3A, ISS and R-ISS stages 1 and 2) in different treatment periods. The 3 years PFS of patients in the before-and after-2013 groups were 31.0 vs. 45.1%, 31.4 vs. 43.5%, 29.7 vs. 46.2%, respectively (p = 0.024, 0.012, 0.001). According to ISS and RISS stages, there were also some differences in OS for non-high-risk patients, with 5-year OS of 44.9 vs. 72.2% and 53.3 vs. 71.5%, respectively (*p* = 0.096, 0.086). However, in high-risk patients (including patients in D-S stage 3B, ISS and R-ISS stages 3), no difference was observed between the two groups in PFS or OS (Table 4).

### Adverse Reactions

A total of 13 patients in the two groups died of non-disease progression. In the before-2013 group, four patients died of severe infection, whereas three patients died in the after-2013 group for the same reason; two patients died of cerebrovascular accident and three patients died of cardiogenic shock in the after-2013 group. Other grade 3/4 hematologic adverse reactions, including neutropenia, thrombocytopenia, and anemia, were not significantly different between the two groups. There was also no significant difference in non-hematological toxicities including asthenia, infection, constipation, diarrhea, pleural effusion and ascites as well as deep venous thrombosis between the two groups (Table 5). However, regarding herpes virus infection and PN, the incidence rate among patients in the after-2013 group was significantly lower than that in the before-2013 group, especially in the incidence rate of grade 2/3 PN, and there was a significant difference between the two groups. Moreover, the incidence of PN in PTD group was 75.5%, which was significantly higher than that in PD, PAD and PCD groups (42.0, 43.9 and 46.6%, respectively) (*p* < 0.001). The overall incidence of PN was 54.5% in the before-2013 group and it was decreased to 43.1% in the after-2013 group (*p* = 0.009), while grade 2/3 PN decreased from 22.5% (before-2013) to 14.4% (after-2013) (*p* = 0.016). In particular, in PTD group, PN at any grade decreased from 82.9% in the before-2013 group to 61.1% in the after-2013 group (*p* = 0.101), and the grade 2/3 PN decreased from 51.4 to 22.2% (*p* = 0.041). The incidence of PN in other treatment groups also decreased slightly, but there was no significant difference.

**TABLE 5:** Adverse reactions.

Adverse events, n (%)	Before 2013 N = 231	After 2013 N = 299	*p*
Hematologic envents (3/4 grade)			
Neutropenia	35 (15.2)	51 (17.1)	0.555
Thrombcytopenia	31 (13.4)	38 (12.7)	0.809
Anemia	17 (7.4)	16 (5.3)	0.343
Non-hematologic envents (all grades)			
Fatigue	64 (27.7)	92 (30.8)	0.443
Infection	52 (22.5)	72 (24.1)	0.672
Constipation	43 (18.6)	45 (15.1)	0.274
Diarrhea	30 (13.0)	52 (17.4)	0.164
Pleural effusion and ascites	11 (4.8)	10 (3.3)	0.407
Herpes zoster	35 (15.2)	22 (7.3)	0.004
Deep vein thrombosis	2 (0.9)	5 (1.7)	0.410
Peripheral neuropathy	126 (54.5)	129 (43.1)	0.009
Grade 1	74 (32.0)	86 (28.8)	0.416
Grade 2/3	52 (22.5)	43 (14.4)	0.016

## Discussion

Proteasome inhibitors as well as immunomodulators are two categories of the most important new drugs for the treatment of MM, of which bortezomib and lenalidomide were the first available drugs in China and bortezomib is currently the main therapeutic agent for newly diagnosed MM patients in China (He et al., 2014; Lu et al., 2014). Bortezomib has been used for newly diagnosed MM patients in China since 2006 for over 12 years. Shifting away from the initial tryout, more Chinese physicians have now become comfortable using these drug-based treatments for MM patients. Through changing the administration methods, adverse drug reactions are greatly reduced and their prevention has been focused. Also, based on the age, performance status and other conditions, patients could receive individualized therapy. So, the improvement of treatment quality based on drug administration methods and patients’ condition poses certain impact on treatment selections, efficacy as well as patients’ survival.

Bortezomib in combination with dexamethasone (PD) serves as the backbone of bortezomib-based treatment regimens for MM. Studies (Davies et al., 2007; Cavo et al., 2010; Moreau et al., 2011; Ludwig et al., 2013; Ludwig et al., 2015; Gentile et al., 2017; Blommestein et al., 2019) have shown that, triplet combination is the preferred regimen if patients have tolerance of drugs, with efficacy superior to the doublet combination but not inferior to the quadruplet combination. The triplet combination is based on the PD regimen with a third drug, such as PAD (combination with doxorubicin) and PCD (combination with cyclophosphamide), which are the most common combinations. Our initial clinical practice suggested that the PCD regimen and the PAD regimen have similar efficacy on patients and could cause adverse reactions alike (He et al., 2014), yet, cyclophosphamide is cheaper and more convenient. PCD regimen, also known as CyBorD, is also recognized in Mayo Clinic and other treatment centers in the United States (Reeder et al., 2009; Ong et al., 2015), and is recommended as the first-line treatment regimen for symptomatic MM patients. This regimen induces a rapid onset of action, with ORR of up to 80% after two cycles of treatment, overall ORR of more than 90%, and CR rate of about 40%. As a result, more than 60% of our patients were treated with the PCD regimen in the after-2013 group, with overall ORR of 93.2% and CR rate of 40.9%. Studies have shown that PTD regimen (PD regimen combined with thalidomide) achieved promising results, with CR/nCR of 30 ∼ 50%, adverse reactions tolerable, and grade 3/4 PN of about 15%, thus it is considered to be one of the best triplet regimens for MM from the economic perspective and adverse reaction concerns (Cavo et al., 2010; Moreau et al., 2011; Ludwig et al., 2013; Huang et al., 2014; Leiba et al., 2014; Gentile et al., 2017; Blommestein et al., 2019). However, based on our practice, PTD is poorly tolerated, with a PN incidence of more than 80%, and nearly 50% of patients developed grade 2/3 PN (He et al., 2014).This may be partly due to the fact that patients in the before-2013 group were all treated with bortezomib by IV, while the incidence and severity of PN are significantly higher in the patients treated with bortezomib by IV compared to that by SC (Moreau et al., 2011; Arnulf et al., 2012; Merz et al., 2015; Xu et al., 2018). The incidence of PN in 18 patients treated with PTD regimen in the after-2013 group was significantly decreased, thereinto, the incidence of grade 2 and higher was 22.2% (4/18), which was similar to a previous study in China (Wang et al., 2016). Therefore, PTD regimen is still worthwhile to try among Chinese MM patient population, although at present, PRD regimen (PD in combination with lenalidomide) is preferred (Mikhael et al., 2013; Wang et al., 2016; Durie et al., 2017; O’Donnell et al., 2018; Ntanasis-Stathopoulos et al., 2019).

For the use of bortezomib, IV was recommended initially. But Moreau, et al. conducted a randomized controlled study MMY-3012 in patients with refractory and relapsed MM, showing that (Moreau et al., 2011; Arnulf et al., 2012) bortezomib could be administrated subcutaneously with similar efficacy as achieved by IV, while significantly reduce the toxicities caused by IV. Moreover, especially in PN, the incidence rate was decreased from 53% (IV) to 38% (SC), as well as grade 2 and higher PN from 41% (IV) to 24% (SC). Subcutaneous administration was started in December 2012, and the results suggested that the PN incidence rate of patients who received bortezomib by IV in the before-2013 group was significantly higher than that in the after-2013 group (54.5 vs. 43.1%), and the incidence of grade 2/3 PN was 22.5 and14.4%. Moreover, we used acupuncture combined with vitamin B12 to treat PN, which significantly relieved this adverse reaction (Han et al., 2017)and enabled MM patients to receive more cycles of treatment. In addition, bortezomib was administrated from twice-weekly in the beginning to once-weekly afterward (Bringhen et al., 2010; de Arriba de la Fuente et al., 2018). Especially in elderly patients, the efficacy and survival of once-weekly dosing were comparable to that of the twice-weekly schedule, but with much less adverse events occurred. In this study, we used weekly regimen for patients over 65 years old with poor performance status since February 2014, which may enable more elderly patients in the after-2013 group to complete more cycles of therapy (Su et al., 2018; Stadtmauer et al., 2019).

When bortezomib was initially used in Chinese patients, it was not covered by National Health Insurance. The price of each cycle of treatment (including four doses of bortezomib) was more than 50,000 RMB. Considering the economic pressure brought by the treatment, many patients would discontinue the treatment after the disease being controlled. However, in August 2013, the China Charity Association together with Xi’an-Janssen Pharmaceutical Co., Ltd. started to provide assistance for patients with newly diagnosed MM to allow them to receive five more cycles of treatment for medication assistance after four treatment cycles. Since bortezomib has been listed in the National Health Insurance in September 2017, patients and their families could no longer consider the drug costs. With our previous clinical experience, we selected the regimen with positive early efficacy, controllable adverse reactions and more acceptable by the patients. We also evaluated the patients to select more individualized treatment plan and strategy, so that patients can complete the whole course of treatment. In the before-2013 group, an average of 3.1 cycles were completed by patients, whereas an average of 5.4 cycles were completed in the after-2013 group. There was no significant difference in ORR, VGPR and better between the two groups, with 90 and 55%, respectively. According to real-world reports, newly diagnosed MM patients treated with bortezomib-based regimens completed an average of 3 and 4 cycles of treatment (Kim et al., 2014; Lu et al., 2014; Liu et al., 2015; Yang et al., 2015), with ORR of 70 – 90% and VGPR and better of 30 – 60%, which was in accordance with the data in this study. However, in this study, more patients achieved CR response in the after-2013 group, with 39.1% in the after-2013 group and 28.6% in the before-2013 group. Therefore, patients who received more cycles of treatment might obtained deeper efficacy, such as CR (Yong et al., 2016; Coriu et al., 2018; Mohty et al., 2018; Remes et al., 2018).

Survival analysis showed that PFS of patients in the after-2013 group was much superior to that in the before-2013 group, with median PFS of 25.5 and 23.3 m, and 3-year PFS of 41.7 and 27.1%, respectively. However, there was no significant difference in OS. Further performed subgroup analysis found that in the after-2013 group, patients aged 65 years and above, patients with non-high-risk D-S, ISS and RISS stages, i.e., patients with stage 1 - 3A and stage 1–2, respectively, had PFS advantage. There was also a certain advantage in OS in patients with non-high-risk ISS and RISS stages (*p* < 0.1), while there was no significant improvement in either PFS or OS in high-risk patients (Palumbo et al., 2015; Zhong et al., 2017). This result suggested that optimizing the treatment quality and increasing the number of treatment cycles failed to overcome the adverse prognosis brought by high-risk factors. Therefore, further optimization of treatment regimens may be required, such as the use of combinations where each drug has high anti-cancer efficacy as well as different mechanisms, like PRD regimen (Mikhael et al., 2013; Durie et al., 2017; O’Donnell et al., 2018; Sidiqi et al., 2018; Ntanasis-Stathopoulos et al., 2019). In addition, we also noted that the patients who responded to treatment in the after-2013 group had significant better PFS whether in patients with PR response and better or with VGPR and better, suggesting that optimizing the treatment regimen and increasing the number of cycles could further improve the PFS result.

## Conclusion

In conclusion, data in this study showed the clinical practice results of bortezomib for newly diagnosed MM patients since it was first applied in China in 2006. In the practical application of bortezomib, Chinese physicians have been familiar with and known well about the efficacy and adverse reactions of bortezomib in clinical use. Also, they consistently sum up experience, so as to adopt optimized and individualized regimens for patients based on their conditions, including the prevention of adverse reactions and the total care for patients, thereby making non-high-risk and elderly patients obtain better remission and PFS. However, for high-risk patients, new combinations with high anti-cancer efficacy as well as different mechanisms might be further explored.

## Data Availability Statement

The raw data supporting the conclusions of this article will be made available by the authors, without undue reservation.

## Ethics Statement

The studies involving human participants were reviewed and approved by the Ethics Committee of the First Affiliated Hospital of Zhejiang University. The patients/participants provided their written informed consent to participate in this study.

## Author Contributions

JH, DH, ZC, and XH contributed to the study design. GZ, HH, and GW conducted the literature search. YZ, YY, and WW acquired the data. JF and LS wrote the article. ZC performed data analysis. All authors gave the final approval of the version to be submitted. All authors read and approved the final manuscript.

## Funding

The study was supported by National Natural Science Foundation of China (81471532, 81770217) and Zhejiang Provincial Natural Science Foundation (LY17H080001, LY18H080001).

## Conflict of Interest

The authors declare that the research was conducted in the absence of any commercial or financial relationships that could be construed as a potential conflict of interest.

## Abbreviations

**Abbreviations**: MM, multiple myeloma; ORR, overall response; CR, complete remission; PFS, progression-free survival; OS, overall survival; VGPR, very good partial response; PN, peripheral neuropathy; IV, intravenous injection; SC, subcutaneous injection.
